# Home-based exercise alters pulmonary function and cellular stress markers in overweight middle-aged men during covid-19 Home quarantine

**DOI:** 10.1186/s13102-023-00673-9

**Published:** 2023-04-20

**Authors:** Bakhtyar Tartibian, Noushin Azadpour, Rasoul Eslami, Sirwan Mohammad Amini Khayat

**Affiliations:** 1 Department of Exercise Physiology, Faculty of Physical Education and Sport Sciences, Allameh Tabataba’i University, Tehran, Iran; 2 Department of Physiotherapy, Ardabil University of Medical Sciences, Ardabil, Iran; 3 Department of Exercise Physiology, Faculty of Physical Education and Sport Sciences, Urmia University, Urmia, Iran

**Keywords:** Exercise, Inflammation, Lockdowns, Pulmonary function, Severe acute respiratory syndrome coronavirus

## Abstract

**Background:**

This study aimed to investigate the effects of a combined home-based exercise program on potential indicators of severe coronavirus disease 2019 (COVID-19) in overweight middle-aged men during home quarantine caused by COVID-19.

**Methods:**

Forty men (aged 45–64 years) were assigned to the exercise (EXE, n = 20) or control (CON, n = 20) groups. A 6-week combined program was carried out three days/week, starting at 20 min per session at 50% maximal heart rate (HRmax) and advancing to 45 min at 70% HRmax. Pulmonary functional and cellular stress biomarkers were measured before and after the training program. Analysis of the covariance (ANCOVA) was used for comparison between the two groups considering the baseline values.

**Results:**

Thirty-six participants (EXE, n = 17; CON, n = 19) completed the research protocol. The EXE group showed post-training improvements in forced vital capacity (FVC), forced expiratory volume in 1 s (FEV1), FEV1/FVC, Vital capacity (VC), and Forced expiratory flow at 25-75% (FEF25-75) compared to the CON group (P < 0. 05). Further, the plasma levels of fibrinogen, Interleukin (IL)-6, Interleukin (IL)-1β, D-dimer, and angiotensin (Ang II) decreased in the EXE group compared to the CON group (P < 0. 05). After six weeks of the training program, leukocyte counts increased in the EXE group compared to the CON group (P < 0. 05). There was a significant positive correlation between body mass index (BMI) with cardiovascular and inflammatory biomarkers other than white blood cells (WBC) in the EXE group (P < 0.05).

**Conclusions:**

The findings suggest that combined home-based exercise during home quarantine improves risk factors for severe COVID-19 in overweight middle-aged men. These improvements were further correlated with changes in BMI. Future research is required to confirm the findings of this study.

## Background

On December 31, 2019, the World Health Organization (WHO) was informed of an outbreak of pneumonia of unknown cause detected in Wuhan City, Hubei Province of China. Then a novel coronavirus was recognized as the cause of pneumonia cases. This pathogen was identified as severe acute respiratory syndrome coronavirus 2 (SARS-CoV-2), and the associated disease was COVID-19. Subsequently, on 11 March 2020, WHO identified the global COVID-19 outbreak as a pandemic. Although the population groups are affected differently by COVID-19 [1], pre-existing conditions such as a sedentary lifestyle, being overweight, and other chronic diseases have been associated with the risk of getting the infection and experiencing more severe symptoms and higher rates of death [2, 3]. There is consensus that SARS-CoV-2 uses the angiotensin-converting enzyme 2 (ACE2) receptor for entry into the cells [4, 5], and due to the expression of ACE2 throughout the body, it can be associated with chronic comorbid conditions. There is also general agreement that severe COVID-19 infection results in an aggravated pulmonary and systemic inflammatory response [6], increased biomarkers of cardiovascular diseases (CVD) [7], hemodynamic instability [8], and exacerbation of the hematological system [9], all of which may lead to multi-organ failure, poor outcomes, and death.

To stop the virus from spreading and prevent the collapse of health services, governments have imposed collective quarantines on the population with varied degrees of rigor. Quarantine, although effective in reducing the person-to-person spread of the disease [10], results in detrimental physiological and morphological adaptations [11]. It changes lifestyle, particularly by lowering involvement in both acute and chronic physical activities (PA) and unhealthy diets. Physical inactivity leads to weight gain, immune and endothelial dysfunction [12], and elevated oxidative stress, all of which compromise overall health and might increase the vulnerability to chronic comorbid conditions [13]. Studies have indicated that young, healthy volunteers confined to a bed for 20 days lose an average of 11% of their heart volume and 28% of their maximal oxygen intake [14]. The blood volume and hemoglobin content also decrease after two to four weeks of detraining [15, 16].On the other hand, the beneficial effects of regular exercise on the immune system are commonly acknowledged, and studies have shown that those who are physically active have a reduced incidence, severity of symptoms, and morbidity from viral infections [17]. Regular exercise improves cardiorespiratory health, strengthens muscles, and reduces the risk of systemic inflammation, which are the processes by which it reduces the symptoms of a variety of acute and chronic morbidities [18]. Studies investigating the association between PA and the risk of COVID-19 infection are currently scarce, however, several studies have reported a positive impact of regular physical activity on comorbidities associated with severe COVID-19 [19, 20]. In line with this, individuals with high cardiorespiratory fitness [21], higher levels of self-reported physical activity [22], and a normal body mass index (BMI) [23] experienced fewer COVID-19-associated hospitalizations and incidence.

Therefore, considering the literature mentioned above, we speculated that pre-exposure conditioning in overweight middle-aged adults might reduce risk factors for developing severe COVID-19 if exposed to the virus. Hence, the present study aimed to investigate the effect of combined home-based exercise training on prognostic markers and indicators of COVID-19, such as inflammation, coagulation, and cardiorespiratory function in middle-aged adults during the quarantine period.

## Materials and methods

### Participants

Participants were recruited via social media and online platforms, such as Telegram, Facebook, Twitter, WhatsApp, and Instagram. Study participants began living in home quarantine in March 2020, and the study protocol started two months after the commencement of quarantine. It needs to be mentioned that, like many other countries, during this period, Iran has implemented several public health measures, including quarantine, stay-at-home orders (in which people were explicitly urged to stay at home as much as possible), isolation, travel prohibitions, closures of the majority of non-essential businesses (including leisure and sports facilities), and closures of schools and universities. Among 75 men recruited into the study, 40 participants met the inclusion criteria for this study. Men were middle-aged (45–64 years old), overweight (BMI between 25 kg/m^2^ and 29/9 kg/m^2^), sedentary (regular exercise < 60 min/wk), and their weight was stable (< 10% body weight change) for at least six months before enrollment. They were nonsmokers, nonalcoholic, free of acute or chronic disease such as CVD or chronic obstructive pulmonary disease (COPD), and not taking medications such as antihypertensive, lipid-lowering, or anticoagulant medications. The study was conducted under the Declaration of Helsinki Ethical Principles (World Medical Association, 2013), and it was approved by the “Iran National Committee for Ethics in Biomedical Research” with the code IR.ATU.REC.1399.061.

### Sample size

Data from the article of Vasić D et al. [24] were used to determine the sample size. After adding up to 30% attrition, a sample size of 40 was recommended with an α = 0.05, an 80% power, and a mean of 10.9 (SD1 = 3.9) and 7.4 (SD2 = 3.8) for EXE and CON groups. After determining the sample size, we first specified the names of all the participants in a list and then assigned a number to each person. The required number was placed in each group using Kendall and Smith’s table of random numbers. Then, participants were assigned to the study groups (Table 1).

### Experimental design

**Fig. 1:**
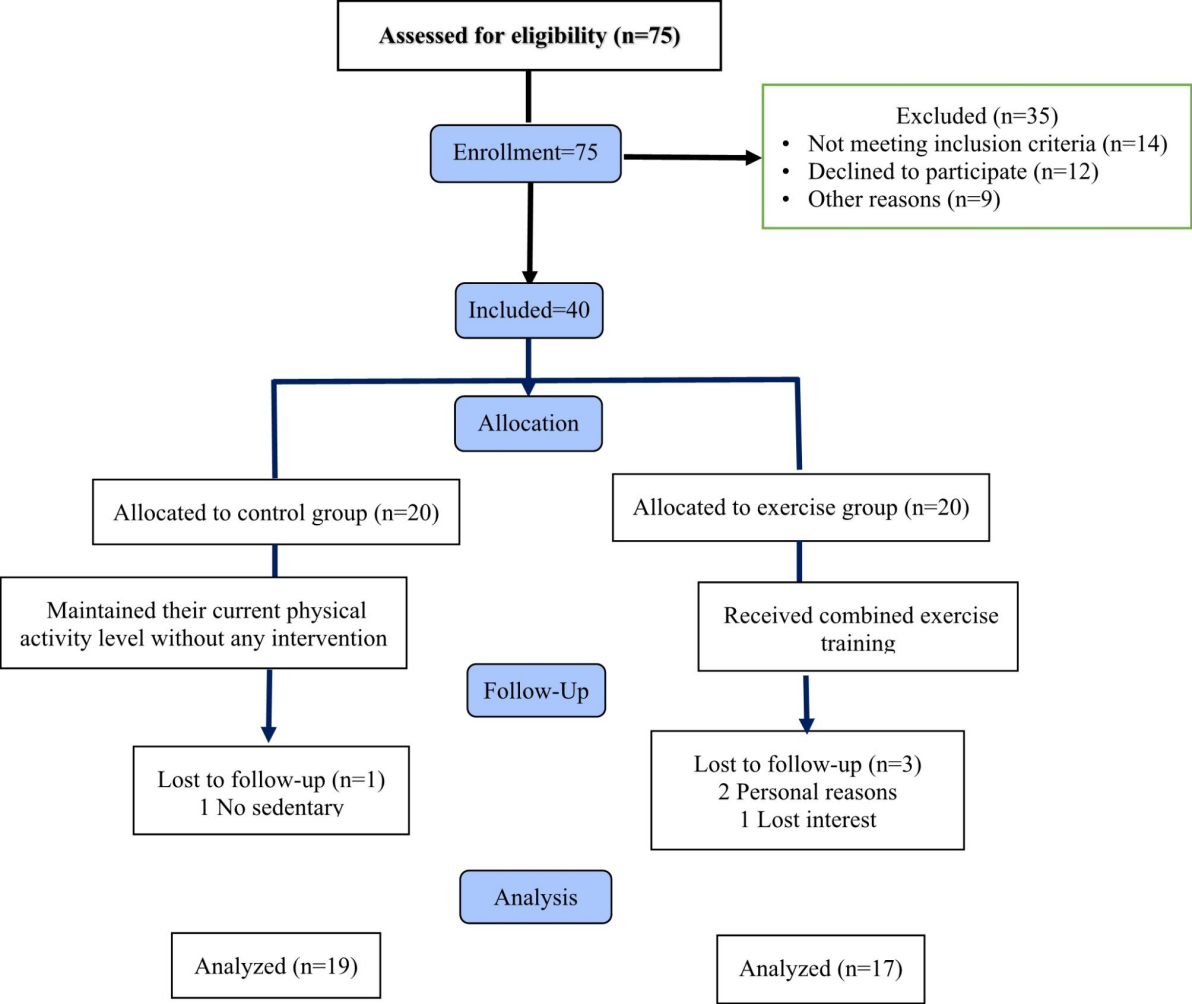
Follow-up diagram throughout the study

In a quasi-experimental, two-group, pretest–posttest design, men who met the inclusion criteria (n = 40) were assigned in a 1:1 ratio to one of the exercise (EXE, n = 20) or control (CON, n = 20) groups. All participants completed three visits. The first visit occurred one week before the first training session via Skype, WhatsApp, and video call. During the first visit, the research purpose and oral explanation of the research process, including blood sampling, exercise protocol, possible benefits, and risks associated with participation in the research, were clearly explained to them. In addition, subjects completed the medical screening form, COVID-19 self-assessment questionnaire, the International Physical Activity Questionnaire (IPAQ), the General Health Questionnaire (GHQ), and the consent form. All the assessment visits took place in the Sports Physiology Laboratory of Allameh Tabatabai University. The second visit (3 days before the start of the training program) was composed of anthropometric measurements, heart rate (HR) and brachial blood pressure (BP) measurements, measurement of peak oxygen uptake (peak VO_2_), collection of blood samples, and nutritional assessment. Participants in the exercise group participated in combined exercise training for six weeks. In contrast, the participants in the control group were instructed to maintain their current physical activity level and dietary habits during the study. Four participants (EXE, n = 3; CON, n = 1) were unable to complete the study protocol and were excluded. As a result, 36 participants (EXE, n = 17; CON, n = 19) completed the research protocol (Fig. 1). All tests were carried out in the morning between 7:00 and 10:00 AM to avoid circadian rhythm variances. The exact order as premeasurements applied for all tests. All participants were asked to abstain from physical activity for 48 h before each test.

### Anthropometrics

All anthropometric tests were performed in the morning after a 12-hour overnight fasting. Height was measured to the nearest 0.1 cm, with a portable SECA stadiometer Model 213 (SECA, Hamburg, Germany) and body weight was measured to the nearest 0.1 kg using electronic weighing scales (Seca 803 electronic scale; Hamburg, Germany). BMI was calculated as weight divided by height squared (kg/m^2^). Waist and hip circumferences were measured using standard approaches and waist-to-hip ratio was calculated.

### Resting heart rate (HR) and blood pressure (BP)

Resting HR was measured using an HR monitor (Polar T31, Finland) after 20 min of seated position. Brachial systolic blood pressure (SBP) and diastolic blood pressure (DBP) were measured on the right arm after 15 min of sitting in a temperature-controlled room using a digital BP monitor (Omron R2 Wrist Blood Pressure Monitor, China).

### Cardiorespiratory fitness and exercise capacity assessments

Cardiorespiratory fitness was assessed by peak oxygen uptake (VO_2_ peak). VO_2_ peak (ml.min^− 1^.kg^− 1^) was measured by performing a modified Bruce protocol. The workload was increased every 3 min by increasing the gradient or the treadmill’s speed until maximal exertion. A 12-lead electrocardiogram continuously recorded HR and rhythm, and subjects reported their rate of perceived exertion (RPE) on the 15-point Borg scale at the end of each stage. Breath-by-breath analysis was used to determine the VO_2_ peak. Therefore, the volume of oxygen consumed (VO_2_) and carbon dioxide produced (VCO_2_) during exercise were calculated using mass flow ventilometry and simultaneously mixing chamber analysis of expired gas fractions (V max E, Sensor Medics, Yorba Linda, CA). Gas analyzers and flow probes were calibrated before each test (gas composition 15% O_2_ and 5% CO_2_). VO_2_ and VCO_2_ were recorded during the final 40 s of each test and at peak exercise and expressed relative to body weight (ml kg^–1^ min^–1^). The exercise test was stopped if there were any exercise-related prohibitions outlined by the American College of Sports Medicine guidelines [25]. Maximal exertion was achieved when the subjects met two of the following criteria: HR > 90% of age-predicted HR _max_ (206 − 0.88 × age) [26], respiratory exchange ratio (RER) > 1.15, RPE of 19 or 20 on the Borg scale [27], or voluntary fatigue. Subjects then walked at low intensity for 7 min to cool down.

Exercise capacity was determined by a standard treadmill test using the Bruce protocol at the Allameh Tabataba’i University (ATU), Tehran. Peak exercise time was notated in minutes, and peak exercise capacity metabolic equivalents (METs) was predicted using standard equations based on peak exercise time [28]. Middle-aged men were verbally encouraged to exercise until voluntary fatigue and lack of symptoms or other signs of cessation. Handrails were allowed if it was essential for safety and balance.

### Dietary assessment

To determine the possible impact of dietary intake on the results of the study as well as any change in the diet throughout the study, the nutrient intake of each subject was assessed via a 3-day food record in the first and last weeks of the training program (over two weekdays and one weekend day) [29]. Dietary intake data were analyzed by dietary analysis software nutrition tracking (Nutrition Tracker Pro 2.0.2 APK).

### Pulmonary function test

All spirometry tests were completed according to the American Thoracic Society / European Respiratory Society (ATS/ERS) guidelines [30] in a sitting position using a portable electronic spirometric device (SP1; Schiller). An experienced technician performed all tests. Briefly, the technician explained and demonstrated the maneuver to all participants. Subsequently, the maneuvers were performed with subjects seated and wearing a nose clip. The device was calibrated after each measurement according to the manufacturer’s recommendations. All spirometry tests were performed during ambient laboratory conditions (mean temperature of 25.7 °C and 31.9% humidity). The spirometry variables measured were vital capacity (VC), forced vital capacity (FVC), forced expiratory volume in 1 s (FEV1), forced expiratory flow at 25-75% (FEF25%‐75%), maximum voluntary ventilation (MVV); peak expiratory flow (PEF). The adopted reproducibility criteria were those from the ATS/ERS guidelines [30]: for test acceptance, the two largest FEV_1_ and FVC values should differ less than 0.15 L; PEF less than 0.5 L; VC less than 0,15 L, and MVV less than 20%. There is no mention of acceptable reproducibility criteria for the FEF_25–75%_ in the ATS/ERS guidelines.

### Blood sampling and assays

Blood samples were collected to determine WBC count and plasma levels of C-reactive protein (CRP), fibrinogen, D-dimer, ferritin, Ang II, IL-6, and IL-1β. Ten milliliters of peripheral venous blood were taken from the antecubital vein after 12 h of overnight fasting. From this sample, 5 ml of whole blood was collected into the Ethylenediaminetetraacetate (EDTA) vial. Blood samples were centrifuged at 3,000 g for 10 min, and the resultant plasma was stored at -80° C until further analyses. CRP was measured quantitatively using the BIONIK kit (Made in Iran, normal range: 4–6 milligrams/liter). To measure fibrinogen levels, the Stago machine (Germany) was used by coagulation method by a Mahsayaran kit of Iranian manufacture. Ferritin blood level was measured according to the results of complexes formed during the antigen–antibody reaction using an ELISA kit (Pishtaz Teb Zaman Diagnostics, Tehran, Iran), and the absorbance was detected at 450 nm using an ELISA reader. D-dimer measurements were performed using a latex agglutination test (Biorex Fars; Iran). The diagnostic specificity and sensitivity of the kit were equal to 98% and 99%, respectively. The plasma level of Ang II was measured by Ang II Enzyme Immunoassay Kit (RayBiotech, Inc Norcross, GA) [31] according to the manufacturer’s instructions. The minimum detectable concentration of Ang II was 2.62 pg/ml. The serum IL-6 and IL1-β levels were detected using commercial human ELISA kits (MultiSciences Biotech, Co., Ltd., Hangzhou, China) according to the manufacturer’s instructions. The IL-6 assay sensitivity was equal to 0.37 pg/ml, and for IL-1β, it was equal to 0.02 pg/mL. The concentration of cytokines was calculated based on standard curves provided with the kits, and results were expressed in pg/ml.

### Exercise training program

In addition to DVDs and step-by-step instructions on performing the exercise protocols, virtual training sessions were provided simultaneously with applications like WhatsApp and Zoom through cellphones and computers. The subjects in the exercise group participated in a progressive home-based training program (including aerobic, resistance, balance, and flexibility) over six weeks, with four sessions per week. Each session was divided into six components: 5–10 min of warm-up, 20–45 min of aerobics, 10–15 min of resistance training, 10 min of balance, 10 min of stretching, and 5–10 min of cool-down exercises [32, 33]. The aerobic exercises were walking, squat jumps, biking, running, or jogging in place, and jumping rope. The time and intensity of the aerobic component were increased from 20 min per session at 50% HR_max_ to 45 min at 70% HR_max_. Participants were controlled with a cardiac monitor (Polar S625X). Participants engaged in resistance exercise using either their own body weight or light free weights. The training sessions involved seven exercises from large muscle groups (including squat, shoulder press, bench press, leg press, leg curl, leg extension, and arm flexion). In resistance exercises, the number of sets increased from 3 sets of 12 repetitions in the first week to 4 sets of 12 repetitions in the second week, five sets of 12 repetitions in the third and fourth weeks, and six sets of 12 repetitions in the last two weeks of the intervention. Overall, the number of repetitions was fixed, and progress in training was applied simultaneously through more sets. The rest period between sets of resistance training was 1 to 3 min. The current research applied resistance through body weight using light dumbbells, elastic bands, and suspension training or total resistance exercises (TRX). Also, increasing the number of sets was one of the conditions for increasing the training intensity. Balance training was based on functional tasks required by middle-aged adults [34]. Balance exercises started from 3 min in the first week and 7 min in the sixth week. The rest between sets was set between 5 and 10 s. At the end and before the cooling down session, participants performed 10 min of stretching exercises designed to improve the flexibility of the major muscle groups; each stretch was sustained between 15 and 30 s to the point of tightness and repeated three times. A 5–10-minute cooling-down was included at the end of the exercise session.

### Statistical analysis

The normal distribution and homogeneity of variance of the data were tested by the Kolmogorov–Smirnov and Levene tests, respectively. Analysis of the covariance (ANCOVA) was used for comparison between the two groups considering the baseline values. Data were analyzed using SPSS 25.0 software (SPSS, Inc., Chicago, IL, USA). The Pearson correlation coefficient was also used to evaluate the relationship between the quantitative variables investigated. The significance level was set at p < 0.05. All data are expressed as means ± standard deviation (SD).

## Results

### Recruitment to trial

A total of 75 healthy sedentary male volunteers were screened for eligibility for the current trial. Of these, 35 subjects (47%) were excluded as they did not meet the inclusion criteria, and the reasons for exclusion are presented in Fig. 1. In addition, 12 subjects were eligible for admission to the trial but declined to participate. Forty (53%) subjects were finally included, and of these, 4 participants (10%) withdrew from the study.

### Participant characteristics

Demographic, body composition, cardiorespiratory fitness measures, and physiological variables before and after the interventions are presented in Table 1. There were no significant differences between the two groups in baseline characteristics. The EXE group showed significant reductions in weight, BMI, SBP, DBP, and resting HR compared to the CON group (P < 0.05) (Table 1). In addition, combined exercise training improved VO_2_ peak and peak exercise capacity in the EXE group compared to the CON group (P < 0.05) (Table 1).

**Table 1:** Demographic and clinical characteristics of participants at baseline

	**Control (n = 17)**		**Exercise (n = 19)**		
**Variable**	**Before**	**After**	**Before**	**After**	***P***
***Demographic variables***					
Age (y)	58.1 ± 6.27	58.1 ± 6.27	57.3 ± 6.39	57.3 ± 6.39	
***Body composition***					
Height (cm)	173.2 ± 3.39	173.2 ± 3.39	173.6 ± 3.67	173.6 ± 3.67	0.34
Weight (kg)	86.0 ± 6.29	87.2 ± 6.46	85.4 ± 8.31	82.7 ± 8.64	< 0.001
BMI (kg/m^2^)	28.6 ± 1.09	28.9 ± 1.09	28.3 ± 1.81	27.4 ± 1.94	< 0.001
***Cardiorespiratory fitness***					
VO_2_ peak (mL/kg/min)	31.7 ± 0.68	30.7 ± 0.51	31.5 ± 0.60	32.8 ± 0.59	< 0.001
Peak Exercise Capacity (Met)	4.6 ± 0.34	4.3 ± 0.32	4.5 ± 0.41	5.5 ± 0.58	< 0.001
***Physiological Variables***					
Brachial SBP (mm Hg)	126.6 ± 2.73	127.4 ± 2.08	127.3 ± 2.35	126.3 ± 2.55	< 0.001
Brachial DBP (mm Hg)	83.0 ± 2.12	83.6 ± 2.08	82.7 ± 2.64	82.2 ± 2.55	< 0.001
Resting HR (bpm)	83.5 ± 2.70	84.4 ± 2.24	82.7 ± 3.55	80.6 ± 3.67	< 0.001

Data are presented as mean ± SD.

BMI, body mass index; VO2 peak, Peak oxygen uptake; SBP, systolic blood pressure; DBP, diastolic blood pressure; HR, heart rate.

*P*, between group differences; ɳ2, partial eta squared indicating effect size.

### Dietary assessment

The subjects’ dietary intakes, including the quality, quantity, and frequency of food consumption, were similar in the two groups. The dietary intakes did not change more than expected over the six weeks of the study (P > 0.05). Subjects did not take any drugs or medications, supplements, and foods known to affect the dependent variables during the study period.

### Pulmonary function

The EXE group showed significant improvements in FVC, FEV1, FEV1/FVC, VC, and FEF25-75 compared to the CON group (P < 0.05) (Table 2). The CON group demonstrated no significant changes in all pulmonary function variables over the six weeks (P > 0.05) (Table 2).

**Table 2:** Comparison of pulmonary function between exercise and control groups before and after the training program

	CON (n = 17)			EXE (n = 19)				
Variable	Before	After	P^a^	Before	After	P^a^	P^b^	ɳ^2^
FVC (L)	4.59 ± 0.71	4.55 ± 0.68	0.054	4.31 ± 31.78	4.96 ± 0.67	< 0.001	< 0.001	-2.21
FEV1 (L)	3.32 ± 0.35	3.27 ± 0.42	0.064	3.48 ± 0.38	3.97 ± 0.27	< 0.001	< 0.001	-2.22
FEV1/FVC (%)	77.00 ± 6.96	75.6 ± 68.22	0.055	75.82 ± 5.95	80.05 ± 7.33	< 0.001	< 0.001	-2.21
PEF (l/min)	570.78 ± 17.34	568.94 ± 18.93	0.244	562.88 ± 15.09	566.41 ± 16.76	0.061	0.106	0.077
VC (L)	4.69 ± 0.61	4.65 ± 0.60	0.055	4.71 ± 0.58	5.11 ± 0.34	< 0.001	< 0.001	0.540
MVV (L)	130.14 ± 16.90	129.74 ± 17.16	0.060	129.95 ± 18.75	130.10 ± 18.37	0.130	0.098	0.81
FEF25-75 (L/S)	3.56 ± 0.38	3.41 ± 0.33	0.059	3.68 ± 0.33	4.05 ± 0.36	< 0.001	< 0.001	-1.50
Data are presented as mean ± SD. FVC, forced vital capacity; FEV1, forced expiratory volume in the first second; PEF, peak expiratory flow; VC, vital capacity; MVV, maximal voluntary ventilation; FEF25-75, forced expiratory flow at 25 and 75% of the pulmonary volume. ^*a*^Within-group difference. ^*b*^Between-group difference. ɳ^2^, partial eta squared indicating effect size.								

### Cardiovascular and inflammatory biomarkers

Cardiovascular and inflammatory biomarkers before and after the 6-week combined exercise training are shown in Table 3. The plasma levels of fibrinogen, IL-6, IL-1β, D-dimer, CRP and Ang II significantly decreased in the EXE group compared to the CON group (P < 0.05). Leukocyte counts after six weeks training program increased in the EXE group compared to the CON group (P < 0.05). In addition, plasma levels of D-dimer and IL-1β increased significantly in the CON group after six weeks (P < 0.05). However, the CON group had no significant changes in other cardiovascular and inflammatory biomarkers (P > 0. 05).

**Table 3:** Comparison of cardiovascular and inflammatory biomarkers between exercise and control groups before and after the training program

	CON (n = 17)			EXE (n = 19)				
Variable	Before	After	P^a^	Before	After	P^a^	P^b^	ɳ^2^
CRP (mg/L)	3/83 ± 0/48	4/00 ± 0/54	0.32	3/61 ± 0/43	2/18 ± 0/58	< 0.001	< 0.001	2.16
Ferritin (ng/mL)	108/21 ± 11/39	109/47 ± 11/60	0.062	106/88 ± 9/82	106/65 ± 10/44	0.431	0.53	0.109
Fibrinogen (mg/L)	298/37 ± 46/42	304/37 ± 41/97	0.051	288/53 ± 59/60	243/71 ± 62/28	< 0.001	< 0.001	2.26
Il-6 (pg/mL)	4/59 ± 0/97	4/93 ± 0/89	0.15	4/45 ± 0/71	3/18 ± 0/57	< 0.001	< 0.001	2.03
IL-1β (pg/mL)	3/56 ± 0/84	3/88 ± 0/74	0.004	3/67 ± 0/70	2/71 ± 0/90	< 0.001	< 0.001	3.18
D-dimer (ng/mL)	261/58 ± 31/31	274/74 ± 30/40	< 0.001	258/76 ± 28/02	222/82 ± 32/80	< 0.001	< 0.001	0.738
Angiotensin II (pg/mL)	16/94 ± 5/61	17/23 ± 5/55	0.061	16/04 ± 6/22	15/80 ± 6/23	< 0.001	< 0.001	0.749
Leucocytes (10^3^ /µL)	5/13 ± 1/10	4/83 ± 1/04	0.36	5/49 ± 1/06	6/48 ± 1/59	< 0.001	< 0.001	-1.05

Data are presented as mean ± SD.

CRP, C-reactive protein; IL-6, interleukin-6; IL-1β, interleukin-1beta.

^*a*^Within-group difference. ^*b*^Between-group difference. ɳ^2^, partial eta squared indicating effect size.

### Associations

Pearson product-moment correlation analysis was used to determine the associations between the studied variables. A significant positive correlation was observed between BMI and cardiovascular and inflammatory biomarkers other than WBC in the EXE group. For each unit increase in BMI, there were increased units in CRP, ferritin, D-dimer, fibrinogen, Ang II, IL-6, and IL-1β. In addition, there was a significant negative correlation between BMI with pulmonary function indices and WBC in the EXE group. For each unit increase in BMI, there were decreased units in pulmonary function indices. A significant negative correlation was found between VO_2 max_ and cardiovascular and inflammatory biomarkers other than ferritin and D-dimer in the EXE group. In addition, there was a significant positive correlation between VO_2 max_ with pulmonary function indices and WBC. Finally, we found a significant negative correlation between peak exercise capacity and cardiovascular and inflammatory biomarkers other than D-dimer in the EXE group. In addition, there were significant positive correlations between peak exercise capacity and pulmonary function and WBC other than FVC in the EXE group (Table 4).

**Table 4:** The correlation between the studied variables during the study

	BMI	VO_2_ max	Peak Exercise Capacity
CRP	0.46*	− 0.45*	-0.40*
Ferritin	0.39*	-0.319	-0.39*
D-dimer	0.22*	-0.233	-0.233
Fibrinogen	0.24*	-0.18*	-0.18*
Ang II	0.16*	-0.11*	-0.11*
IL-6	0.45*	-0.42*	-0.42*
IL-1β	0.32*	-0.27*	-0.27*
FVC	-0.14*	0.54*	0.54
FEV1	-0.36*	0.39*	0.39*
FEV1/FVC	-0.41*	0.15*	0.15*
PEF	-0.30*	0.23*	0.27*
VC	-0.14*	0.14*	0.14*
MVV	-0.33*	0.31*	0.31*
FEF	-0.44*	0.31*	0.31*
WBC	-0.22*	0.11*	0.11*

BMI, body mass index; CRP, C-reactive protein; Ang II, angiotensin II; IL-6, interleukin 6; IL-1β, interleukin 1 beta; FVC, forced vital capacity; FEV1, Forced expiratory volume in the first second; PEF, peak expiratory flow; VC, vital capacity; MVV, maximum voluntary ventilation; FEF, forced expiratory flow; WBC, white blood cells.

P < 0.05

## Discussion

This study investigated the effect of combined home-based exercise training on some COVID-19 severity indicators in sedentary overweight middle-aged men during home quarantine. The primary findings of the present study were that six weeks of home-based exercise training substantially improved the inflammatory, cardiovascular, and respiratory indicators in overweight middle-aged men. Also, these changes were associated with significant alterations in body weight.

### Markers of inflammation

Findings from several cross-sectional [35, 36] and prospective studies [37, 38] have associated regular physical activity with decreases in systemic inflammation, especially in obese individuals with chronic inflammatory conditions. It is generally recognized that regular exercise has a significant impact on the normal functioning of the immune system. The general consensus is that immune regulation and exercise are connected to, and through its impact on leucocytes, red blood cells, cytokines, etc., exercise training alters immunological regulation [39]. Also, via its anti-inflammatory properties, regular exercise may decrease the incidence of viral infections, the duration of infection, and the mortality rate [40, 41]. In the present study, the plasma levels of IL-6, IL-1β, and CRP significantly decreased in the EXE group compared to the CON group. These results are in accordance with earlier findings showing that sustained physical activity is associated with decreased markers of inflammation [42, 43]. In addition, at six weeks of the intervention, leukocyte counts significantly increased to baseline in the EXC group. These inflammatory markers have already been connected to the severity of the disease in COVID-19 patients [44] and have prognostic importance, with higher levels associated with worse outcomes [45]. No previous research has investigated pre-exposure conditioning in middle-aged adults; however, the exercise-induced decreases in markers of inflammation might be related to the reduced risk factors for developing severe COVID-19 if exposed to the virus. Also, it is probable that the reported effects were connected to the changes in body composition because these alterations were correlated with changed BMI and body fat percentage.

### Cardiovascular and coagulation markers

Numerous studies have discovered a connection between elevated procoagulant markers d-dimer [46] and fibrinogen [47], and cardiovascular risk factor Ang II [48], compromised coagulation function, disease severity, and a more significant mortality rate in viral infections. To determine if regular exercise interacts with coagulopathy markers and cardiovascular risk factor Ang II in inactive, overweight middle-aged men during home quarantine, we looked at the variation in these levels in response to the home-based exercise intervention. Regular exercise routines result in favorable coagulation [49, 50] and Ang II responses [51], as shown by our research and prior interventional investigations. This improvement may be attributable to training-induced adaptations in the coagulation and fibrinolytic systems. Accordingly, regular exercise training appears to help the coagulation and fibrinolytic systems during home quarantine. It may lower cardiovascular risks in this population, and these mechanisms may help explain some of the positive cardiovascular benefits of home-based exercise training. Additionally, given that these adjustments were associated with altered BMI and body fat %, it is likely that the reported effects were due to changes in body composition.

### Pulmonary function

Regular exercise is associated with improved lung function, significantly improving health-related quality of life [52]. Nevertheless, long-term homestays may result in physical inactivity and a sedentary lifestyle, which may worsen lung function [53] and induce a more profound loss in health-related quality of life [54]. However, to date, there is no proof that physical exercise during the COVID-19 period of home confinement affects pulmonary function. In the present study, six weeks of home-based exercise training induced significant improvements in FVC, FEV1, FEV1/FVC, FEF25-75 and VC than the sedentary CON group. In the present study, it seems, home-based exercise training protocol strengthens the endurance of respiratory muscles, particularly the diaphragm, and averts respiratory muscle fatigue in overweight middle-aged men during home confinement. As a result, it is possible to suggest combined home-based exercise intervention to enhance pulmonary function, which is crucial for preserving a health-related quality of life during a COVID-19-related home quarantine.

### Clinical characteristics

It is hypothesized that decreased physical activity levels below the daily target of 7500–10,000 steps may worsen inactivity-related health issues and raise the risk of numerous harmful health conditions [55]. In the study by Dergaa et al. (2022), marked reductions in physical activity levels, step counts, and calories/day were observed in response to the expansion in the COVID-19-induced detraining period during home quarantine [11]. The authors concluded that variables reducing physical activity levels during home confinement include routine changes, elevated stress, and enhanced anxiety (REF 100). These detrimental adaptations were, however, successfully reversed by the higher dose of physical activity during the month following confinement. In the present study, our results demonstrated significant post-exercise improvements in body weight, BMI, SBP, DBP, resting HR, VO_2_ peak, and peak exercise capacity in this population, suggesting that increased levels of physical activity may lead to improvements in health-related outcomes during the COVID-19-induced detraining period during home quarantine.

### Limitations

This study has several limitations. An important limitation of this study was the small sample size, and we could not include other relevant parameters to this context, like the antioxidant or oxidative stress biomarkers. In addition, this study focused only on overweight middle-aged men. A quasi-experimental design was used in this study. D-dimer is a costly parameter that is inaccessible to many research methods; thus, our work is the first to look into it in this context. There is a conclusive demand for additional studies in larger cohorts with prolonged follow-up periods to investigate the effects of home-based exercise interventions on biochemical and hematologic markers during a COVID-19-related home quarantine.

## Conclusions

In conclusion, the current study indicates that six weeks of combined home-based exercise during home quarantine, decreases the blood levels of markers involved in the severity of COVID-19 and improves pulmonary function in middle-aged, overweight men. Home-based exercise training caused significant reductions in the levels of pro-inflammatory mediators, suggesting an anti-inflammatory effect. The intervention also improved pulmonary function and positively influenced coagulation and cardiovascular indicators. These changes were correlated with altered BMI and may positively impact health-related quality of life in this population. Overall, the results indicate that home-based exercise training during quarantine reduces risk factors for severe COVID-19 in middle-aged men. From a clinical perspective, biological indicators of cellular stress should be regarded as significant prognostic markers of the disease. Averting the process of increase in the prognostic markers of Covid-19 during home quarantine through a combined exercise protocol in middle-aged men can be considered a non-pharmacological preventive approach. The findings advance our understanding of the COVID-19 biomarkers and are worthy of greater clinical attention and future systematic research.

## Data Availability

The datasets used during the current study are not publicly available due to confidential information about the participants but are available from the corresponding author on reasonable request.

## Declarations

### Manuscript writing

Artificial intelligence, was not used at any stage of writing the manuscript.

## Abbreviations

PA: Physical activity
HRmax: Maximal heart rate
ANCOVA: Analysis of the covariance
BMI: Body mass index
COVID-19: Coronavirus disease 2019
SARS-CoV-2: Severe acute respiratory syndrome coronavirus 2
WHO: World Health Organization
CVD: Cardiovascular diseases
ACE2: Angiotensin-converting enzyme 2
IL-6: Interleukin-6
TNF-α: Tumor necrosis factor alpha
CRP: C-reactive protein
WBC: White blood cell
COPD: Chronic obstructive pulmonary disease
Peak VO_2_: Peak oxygen uptake
SBP: Systolic blood pressure
DBP: Diastolic blood pressure
RPE: Rate of perceived exertion
METS: Metabolic equivalents
RER: Respiratory exchange ratio
VC: Vital capacity
FVC: Forced vital capacity
FEV1: Forced expiratory volume in 1 s
FEF25%-75%: Forced expiratory flow at 25‐75%
MVV: Maximum voluntary ventilation
PEF: Peak expiratory flow
TRX: Suspension training or total resistance exercises
1RM: One-repetition maximum
SD: Standard deviation
